# The DAWN Alzheimer's Research Study: A multi‐site collaboration with enriched AD phenotype clinical data for the Alzheimer's Disease Sequencing Project‐ADSP

**DOI:** 10.1002/alz70855_106589

**Published:** 2025-12-24

**Authors:** Pedro R Mena, Andrew F Zaman, Michael L Cuccaro, Katalina F. McInerney, Larry D Adams, Jenny Chavez, Christiane Reitz, Giuseppe Tosto, William S Bush, Jonathan L Haines, Allison M Caban‐Holt, Goldie S Byrd, Brian W Kunkle, Jeffery M Vance, Adesola Ogunniyi, Rufus O Akinyemi, Margaret Pericak‐Vance

**Affiliations:** ^1^ University of Miami Miller School of Medicine, Miami, FL, USA; ^2^ John P. Hussman Institute for Human Genomics, University of Miami Miller School of Medicine, Miami, FL, USA; ^3^ Gertrude H. Sergievsky Center, Taub Institute for Research on the Aging Brain, Departments of Neurology, Psychiatry, and Epidemiology, College of Physicians and Surgeons, Columbia University, New York, NY, USA; ^4^ Department of Population and Quantitative Health Sciences, Institute for Computational Biology, Case Western Reserve University, Cleveland, OH, USA; ^5^ Cleveland Institute for Computational Biology, Case Western Reserve University, Cleveland, OH, USA; ^6^ Wake Forest University School of Medicine, Winston‐Salem, NC, USA; ^7^ College of Medicine, University of Ibadan, Ibadan, Oyo, Nigeria; ^8^ John P. Hussman Institute for Human Genomics, University of Miami, Miami, FL, USA

## Abstract

**Background:**

The DAWN Alzheimer's Research Study is an international multi‐site initiative to build a resource aimed at increasing genetic ancestry representation in Alzheimer disease (AD) studies to advance our ability to leverage all genetic variation in understanding and treating AD for everyone. Consisting of 4,000 Americans with African ancestry (AAA) and 4,000 Latin Americans (LA) from four US sites and 5,000 Indigenous Africans (IA) from nine African countries, this resource of 13,000 individuals is collecting extensive clinical, social, behavioral and environmental information along with genetic and biomarker data.

**Method:**

Participants are ascertained from both clinical (e.g., hospital, physician practices, etc.) and community settings (e.g., community centers, home visits, etc.) resulting in a range of clinical classifications (AD/dementia [AD/DEM], AD Related Dementia [ADRD], mild cognitive impairment [MCI], and no cognitive impairment [NCI]). All participants are administered a standard clinical protocol which includes: clinical and family history, neuropsychological screeners and assessments, functional assessment, neurobehavioral measures and epidemiological assessments. All measures were developed to support subsequent harmonization with cohorts in the Alzheimer's Disease Sequencing Project.

All clinical data is collected and compiled in Redcap where it undergoes rigorous quality control processing. Resulting clinical data are then processed using a combination of decision tree‐based algorithms and reviewer‐based adjudication methods. Workflows are adapted for our US and African sites, and aligned to a standard set of research diagnostic criteria.

**Result:**

In our first two years we have ascertained 2,198 (IA) individuals (58% Female, mean age =74.5 years, mean education = 7.4 years), 1,616 AAA (81% Female, mean age=71.6 years, mean education = 14.3 years), and 1,599 LA (76% Female, mean age=72.2 years, mean education = 10.9 years).

Clinical research diagnoses are distributed as follows: (a) IA ‐ 51% NCI, 38% AD, and 11% ADRD and MCI; (b) AAA ‐ 58% NCI, 8% AD, and 34% ADRD and MCI; (c) LA ‐ 43% NCI, 19% AD, and 38% ADRD and MCI.

**Conclusion:**

Our ongoing efforts in the DAWN Study have been productive. To date the project has ascertained, enrolled, and processed more than 5,000 participants. This collaborative project will serve as a catalyst for identifying and understanding genetic risk for all populations.

